# Generalized Dermatitis Caused by *Staphylococcus aureus* in the European Bison

**DOI:** 10.1155/crve/6559440

**Published:** 2025-12-28

**Authors:** Betina Boneva-Marutsova, Plamen Marutsov

**Affiliations:** ^1^ Department of Veterinary Microbiology, Infectious and Parasitic Diseases, Faculty of Veterinary Medicine, Trakia University, Stara Zagora, Bulgaria, uni-sz.bg

**Keywords:** *Bison bonasus*, case report, dermatitis, *Staphylococcus aureus*, wildlife health

## Abstract

Bacterial dermatitis in animals caused by staphylococci is considered secondary and often results from prior conditions related to environmental factors (like ectoparasites, skin injuries, temperature, and humidity) and internal factors (such as deficiency conditions, immunological dysfunction, and underlying diseases). The case represents a generalized dermatitis in a female European bison calf caused by *Staphylococcus aureus*. Clinically, dermatitis with symptoms of alopecia and crusting was observed. The skin appeared scaly, dry, and rough, covered with thick crusts and showed no signs of itching. Upon the removal of the crusts, inflamed, swollen, and oozing lesions were revealed underneath. Based on the laboratory results, the definitive therapy was initiated. After a lengthy treatment course, the bison′s condition improved, and new fur covered its body. This case emphasizes the necessity of targeted measures for timely etiological diagnosis. Furthermore, careful health monitoring and proactive disease management are essential for wildlife species, which rely directly on human actions for their preservation.

## 1. Introduction

The skin operates as a complex ecosystem, providing a multicomponent habitat with its folds, invaginations, and lesions that support a diverse range of microorganisms. The composition of the skin microbiota varies by body site and includes different types of organisms, such as bacteria (like *Proteobacteria*, *Corynebacterium*, and *Staphylococcus* species), fungi (such as *Malassezia*), and viruses (for instance, Capripox) [1]. A healthy skin microbiota is crucial for maintaining skin health. It occupies sites where pathogens could adhere and produces a variety of substances that inhibit pathogen growth.

Numerous pathogens, including bacteria, viruses, fungi, and parasites, can induce skin infections in domestic and wild ruminants. Commonly encountered infections include dermatophilosis (rain scald), ringworm (dermatophytosis), lumpy skin disease, sheep pox, goat pox, as well as parasitic infestations like mange, lice, and warble fly (genus *Hypoderma*). Other prevalent conditions in large ruminants include digital dermatitis in dairy cattle and papillomatosis [2–5].

European bison (*Bison bonasus*), also called wisent, generally demonstrate robust health and a pronounced capacity to withstand many common diseases. This resilience is likely attributable to their immune system, along with the fact that they are not prone to diseases that commonly affect domestic cattle. However, when the immune system becomes compromised, certain pathogens may exploit this opportunity to induce disease. Documented cases of skin diseases, such as bovine papillomatosis and digital dermatitis, have been observed in captive European bison [6, 7].

It is important to note that bacterial skin diseases in both domestic and wild ruminants are frequently underdiagnosed. Specific risk factors that compromise the skin′s barrier can allow opportunistic pathogens to proliferate, potentially leading to the onset of disease. Disruption of skin integrity can lead to various skin and soft tissue infections, such as impetigo, cellulitis, folliculitis, furunculosis, and others [8, 9]. A study by Abrahamian and Goldstein [10] reveals that dermal wounds can be colonized by a combination of aerobic bacteria, primarily *Staphylococcus* and *Streptococcus* spp., as well as anaerobic bacteria such as *Corynebacterium* and *Trueperella* spp. Among the most commonly isolated bacterial pathogens responsible for skin diseases in animals are staphylococci. Research on wild animals such as red deer, roe deer, and squirrels has confirmed that staphylococci are causative agents of dermatitis, with *Staphylococcus aureus* being the most frequently isolated species. It possesses a complex arsenal of virulence factors related to its ability to adhere to host tissues, secrete a wide range of toxins (alpha‐toxin, Panton‐Valentine leukocidin/PVL, superantigens, such as toxic shock syndrome toxin‐1/TSST‐1, and exfoliative toxins), and form biofilms [8, 11, 12].

## 2. Case Description

European bison were successfully reintroduced to the Rhodope Mountains in 2013. Since then, their population has shown a gradual increase.

### 2.1. Case History

On August 13, 2019, the herd made its way down from the mountain in the *Studen Kladenets Game Reserve* area where they were first brought. Almost all animals were found to have a high tick infestation. Two of the females (one with a calf about 3 months old) showed weakness and an unsteady gait. The condition progressed to a lack of coordination and recumbency. According to the local veterinarian, the most likely diagnosis is tick paralysis. All animals in the herd were treated pоur on with deltamethrin (Spotinor Norbrook), and the sick ones were also treated injectable with ivermectin (Kepromec Kepro) and tetracycline antibiotic (Tetravet Ceva). The condition of all animals is gradually improving.

The female calf named Nadezhda, born on May 11, 2019, with a pedigree number 14407, has severe hair loss and scabs. Attempts to treat with ivermectin did not yield results, so a sample of scraped material (crusts and hairs) was obtained for laboratory testing. The result obtained by the laboratory was negative for scabies and ringworm. The condition progresses to involve large parts of the body, which corresponds to a decreased appetite and subsequent weight loss.

**Figure 1:**
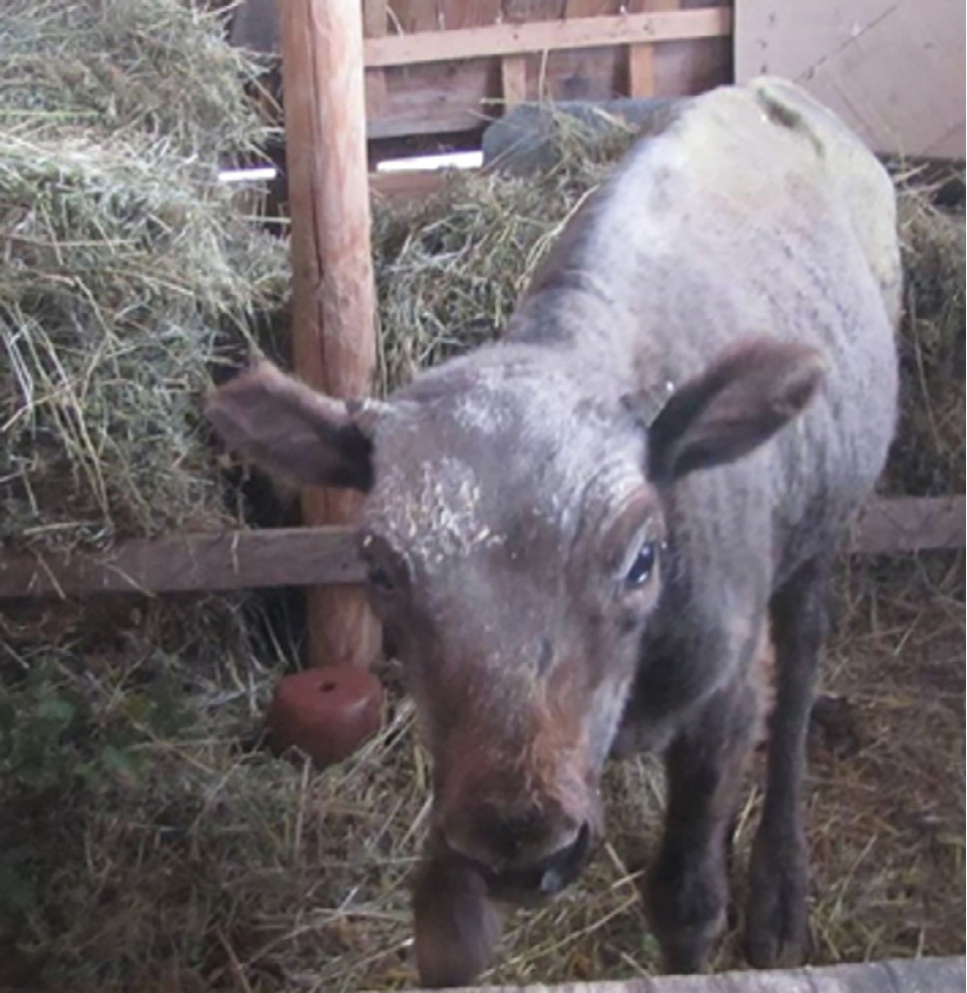
A bison calf in the barn with skin changes exhibiting hair loss, crusting, and exfoliation in the head area.

In early November, reduced solar radiation lowers temperatures as the days grow shorter. The atmospheric conditions contribute to frequent fog and rainfall. During the regular rounds to inspect the herd, the bison calf was found in the mud, unable to stand. The adult herdmates surrounded and guarded it. With great effort, the calf was carefully placed in a car and then moved to a barn. There, it was warmed and fed by hand (Figure 1). On November 23, 2019, a veterinarian from the Faculty of Veterinary Medicine at Trakia University visited the site.

### 2.2. Physical Examination

**Figure 2:**
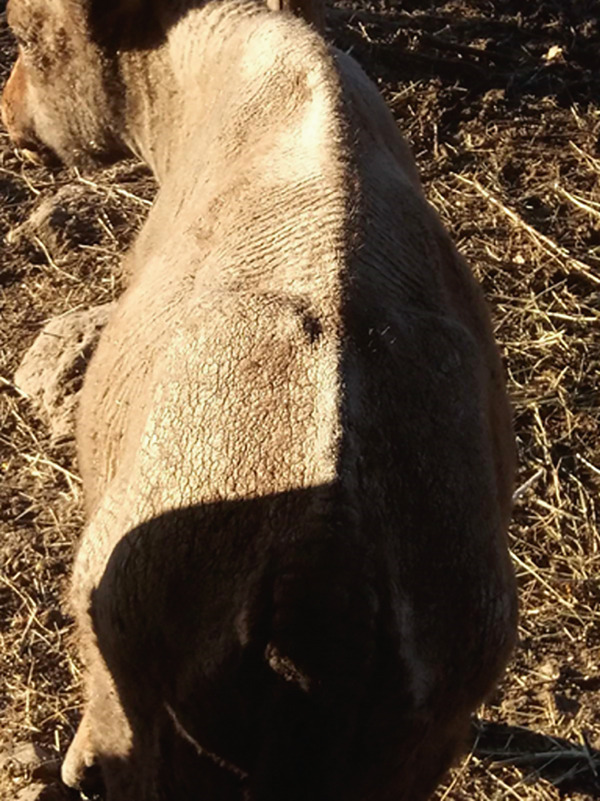
Exuberant cutaneous lesions exhibiting generalized alopecia, with thick surface crusts located on the back and rump (before treatment).

During the general examination, normal temperature, pulse, and respiration were observed, and the calf was alert and interested in both food and its surroundings. Significant changes were observed on the skin, described as generalized dermatitis with alopecia and crusting. The condition affects multiple regions of the integument, such as the face, neck, back, flanks, limbs, and perineal area. The skin appears flaky, dry, rough, covered with thick crusts, and the lesions were not found to cause itching (Figure 2). Upon removal, inflamed, edematous, and oozing lesions are revealed underneath.

## 3. Diagnostic Methods, and Laboratory Findings

No bacteriological investigations were conducted previously. During the visit, swab samples were collected for bacteriological investigations from the lesions after the removal of crusts by using a sterile scalpel blade and tweezers. Samples were transported to the laboratory using a cool box filled with cool packs.

The specimen was cultured using conventional microbiological techniques. Inoculations were performed in liquid broth medium (Tryptone Soy Broth, Oxoid) and on solid nutrient media (Blood Agar Base and MacConkey agar, Oxoid). Streaking was carried out using the four‐quadrant streak plate method, and cultures were incubated at 37°C for 48 h [13].

After 24 h of incubation, two distinct staphylococcal isolates were recovered. Colony density was classified as high, with numerous colonies observed across the first three quadrants. The predominant growth consisted of round, raised, opaque colonies measuring 1–2 mm in diameter, with a characteristic yellow to golden pigmentation. On blood agar, these colonies exhibited clear zones of *β*‐hemolysis, indicative of pronounced metabolic activity and potential virulence. They were later determined to be coagulase‐positive. In contrast, the other isolate displayed single, small, white, opaque, flat, and nonhemolytic colonies, also 1–2 mm in diameter. The tube coagulase test for this strain was negative. Microscopic examination revealed that both isolates were composed of similar Gram‐positive spherical cells arranged in tetrads or clusters. Further identification of the isolates was subsequently performed using the Vitek 2 system (BioMérieux Inc., Durham, NC) with GP ID cards. Vitek 2 GP card analysis confirmed the isolates as *S. aureus* and *Staphylococcus epidermidis*, respectively. The antibiotic susceptibility test conducted with the AST‐P506 card of the Vitek 2 system revealed the susceptibility profile for the isolated *S. aureus* strain, as presented in Table 1.

**Table 1:** Antimicrobial susceptibility profile of the *S*. *aureus* (AST‐P506 card).

**Antimicrobial**	**MIC (*μ*g/mL)**	**Interpretation**
Amoxicillin	≤ 4	S
Benzylpenicillin	≤ 2.0	S
Cefotaxime	≤ 0.5	S
Ceftriaxone	≤ 0.25	S
Chloramphenicol	≤ 8	S
Erythromycin	≤ 0.5	S
Ofloxacin	≤ 0.25	S
Tetracycline	> 16	R
Vancomycin	≤ 1.0	S

*Note:* Testing according to the EUCAST. The interpretation of the results of the antibiotic sensitivity test is based on established breakpoints for systemic use.

Abbreviations: R, resistant; S, sensitive.

### 3.1. Treatment

Following a definitive diagnosis, appropriate therapy was promptly commenced. The treatment of the bison calf was adjusted based on the results of the in vitro susceptibility tests. For this purpose, amoxicillin was administered at a dose of 15 mg/kg of body weight via the intramuscular route. The initial attempts at injection were unsuccessful due to strong muscle tension and difficulties in administering the correct dosage. As a result, the route of administration was changed, and the calculated daily dose was given orally. To facilitate this, food baits made with apples and mushrooms were prepared. The treatment duration was until the skin lesions healed (> 45 days) and hair regrowth appeared. Injectable vitamins (Multivitamins Pantex Holland) at the recommended dosage were applied by the subcutaneous route. Additionally, the treatment included the use of a 4% chlorhexidine solution used to soften and remove necrotic tissue, crusts, and debris. An oral supplement containing zinc (2 mg/kg/day for at least 3 weeks) and zinc oxide applied as a topical cream accompanied the treatment.

### 3.2. Outcome

**Figure 3:**
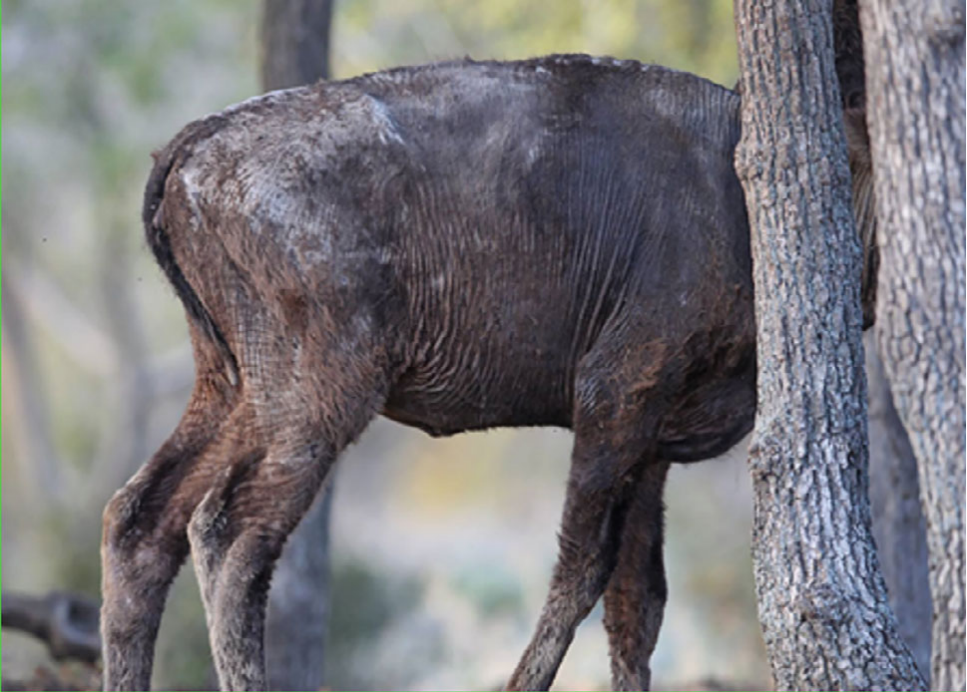
Skin condition shows improvement in 3–4 weeks after the start of treatment, with crusts disappearing and visible initial hair growth becoming apparent.

The calf′s condition demonstrated gradual clinical improvement after initiation of antimicrobial therapy. Reepithelialization was observed, with the defect gradually closing. In milder lesions, superficial pigmentation and hair regrowth began after 20–25 days (Figure 3). Larger and deeper lesions took an additional 20–30 days to heal completely. Full recovery of the skin and fur takes nearly 3 months.

## 4. Discussion

The most skin diseases in wild and domestic animals are caused by a variety of pathogens, including fungi, parasites, viruses, and bacteria [14]. Bacterial dermatitis in European bison in captivity or the wild is rarely reported. Reports of digital dermatitis caused by *Treponema* bacteria have been documented in six females and four males at Berne Animal Park [15]. This case highlights the potential occurrence of staphylococcal dermatitis in wisent calves and underscores the importance of collecting appropriate samples for a thorough clinical workup of skin diseases in animals.

The skin changes observed during examination support a presumptive clinical diagnosis of dermatitis; however, accurate identification of the cause and appropriate treatment require in‐depth laboratory testing. In this particular case, a skin infection in a bison calf was found to be associated etiologically with two types of staphylococci. Among bacterial pathogens, staphylococci are the most common cause of skin problems. Integumentary disorders due to staphylococcal infection in livestock species include folliculitis, impetigo, pyaemia, mastitis, exudative epidermitis, periorbital eczema, furunculosis, scalded skin syndrome, and pyoderma [8, 16]. In this case, considering the uncertain role of *S. epidermidis* in disease and its predominantly commensal nature, we assumed that *S. aureus* played the primary role in the development of dermatitis.

Most staphylococcal infections are opportunistic, arising secondary to other diseases or a compromised host immune system. Damage to the skin barrier allows commensal and pathogenic bacteria to proliferate and penetrate deeper skin layers [17, 18]. Bacterial dermatitis may also develop in response to ectoparasite infestations, including fleas, lice, mites, and ticks [19]. A previously negative result for scabies and ringworm could occur due to improperly selected and stored samples, treatment with ivermectin, or limitations in the laboratory′s diagnostic capabilities. Other possible disease risks may be related to immune system disorders, which can be caused by genetic factors. Due to the limited number of individuals, the European bison is considered a highly inbred species, making it more susceptible to various infectious diseases [20]. These factors, together with other contributing elements such as maternal illness, moisture, traumatic injuries, and stress, form the basis for the development of staphylococcal dermatitis. In contemporary science, different risk factors are regarded as component causes that act together to form a sufficient cause capable of inducing disease [21].

In this case, the absence of timely and appropriate therapy, attributable to an initial failure to establish an accurate etiological diagnosis, resulted in over 2 months of disease progression, leading to chronic, disseminated dermatological lesions (affecting the whole body) and systemic manifestations including weight loss, inappetence, and lethargy. In‐depth laboratory investigations, including bacteriological culture and antimicrobial susceptibility testing, are essential to guide the selection of effective therapeutic agents against bacterial pathogens, particularly in view of the prevalence of multidrug‐resistant strains. The results of such testing enable the initiation of definitive therapy, thereby optimizing treatment efficacy and reducing the risk of further resistance development [22]. The isolate was found to be resistant only to tetracyclines, which are among the commonly used classes of antimicrobials in productive animals. This may pose a risk of resistance spreading to other bacteria, as resistance genes are shared via mobile genetic elements, such as plasmids and transposons [23]. The drug of choice, amoxicillin, is distributed well in the body and achieves effective concentrations in the skin, making it a suitable treatment agent. It is also recommended as a first‐line antibiotic for canine staphylococcal dermatitis [24]. The duration of antimicrobial therapy for bacterial skin infections should be individualized based on the severity of clinical signs, the extent and depth of lesion involvement, and the patient′s general condition. In companion animals with deep tissue integumentary diseases, full resolution may take from 4–6 weeks or even longer [24–26].

*S. aureus* exhibits phenotypic heterogeneity, existing in metabolically active and dormant states, and is capable of forming biofilms that provide structural and chemical protection of the bacterial cells. Dormant cells exhibit antibiotic tolerance due to their minimal metabolic activity, whereas biofilms hinder drug penetration and immune clearance. These factors also necessitate prolonged antimicrobial therapy to eradicate persistent bacterial populations and prevent recurrence of infection [27]. Long‐term antibiotic use can increase the risk of antibiotic resistance and lead to drug toxicity. Although generally well tolerated, amoxicillin may cause gastrointestinal symptoms such as nausea, vomiting, and diarrhea [28, 29]. However, none of these symptoms were observed in the wisent calf.

Topical antimicrobial agents are widely available in veterinary practice; however, their efficacy in ruminants remains poorly studied, with most research focused on dogs and horses. Despite this, topical antiseptics are effective against pathogens responsible for bacterial and fungal skin infections and are commonly employed in cattle to manage bovine digital dermatitis and dermatophytosis (ringworm) [30, 31].

Due to the delicate nature of the skin, careful application of topical treatments is essential to avoid further tissue damage, in accordance with human medical guidelines [32, 33]. In the management of bacterial dermatitis, the therapeutic protocol included debridement followed by irrigation with a 4% chlorhexidine solution, performed three times at 7–10 day intervals. This approach aims to reduce bacterial burden, remove necrotic tissue, and optimize conditions for wound healing.

Oral zinc supplementation has been explored as an additional therapeutic strategy in dermatology due to its vital roles in keratinocyte proliferation, immune regulation, antioxidant defense, and wound healing. In cases of acne vulgaris, zinc demonstrates anti‐inflammatory properties and suppresses the growth of *Cutibacterium acnes*. In hidradenitis suppurativa, zinc has been linked to a reduction in lesion recurrence and control of inflammation. Additionally, zinc enhances epithelialization and collagen synthesis in chronic wounds and ulcers [34]. There is strong evidence that zinc supplementation enhances immune function and contributes to the prevention of foot rot in cattle and sheep, bovine digital dermatitis, and facial eczema in sheep [35–37].

## 5. Conclusion

Skin diseases in wild animals are challenging to diagnose and treat. This case underscores the importance of laboratory testing as a cornerstone of responsible veterinary care, particularly in cases of bacterial infections that necessitate prolonged antibiotic therapy. Targeted use based on antimicrobial susceptibility data will reduce the blind use of antibiotics and thereby reduce the risk of selecting for resistant bacterial pathogens. Rigorous health monitoring is essential for preventing infectious diseases and ensuring the success of reintroduction programs. For the vulnerable European bison population, preserving each individual is critical for the species′ survival. Effective collaboration with veterinary specialists is therefore indispensable to achieving better health outcomes and conservation success.

## Consent

Written informed consent was obtained from the owner for diagnostic testing and publication of this case report.

## Disclosure

This manuscript is an earlier version of the preprint presentation in https://preprint.org/ with the following link: https://www.preprints.org/manuscript/202508.1530/v1.

## Conflicts of Interest

The authors declare no conflicts of interest.

## Funding

This study was supported by Trakia University, 10.13039/501100010200, (BG 123024538).

## Data Availability

The data that support the findings of this study are available from the corresponding author upon reasonable request.
